# Disease-specific autoantibody production in the lungs and salivary glands of anti-synthetase syndrome

**DOI:** 10.3389/fimmu.2024.1265792

**Published:** 2024-06-13

**Authors:** Masaru Takeshita, Katsuya Suzuki, Maho Nakazawa, Hirofumi Kamata, Makoto Ishii, Yoshitaka Oyamada, Hisaji Oshima, Satoshi Usuda, Kazuyuki Tsunoda, Tsutomu Takeuchi

**Affiliations:** ^1^ Division of Rheumatology, Department of Internal Medicine, Keio University School of Medicine, Shinjuku, Japan; ^2^ Division of Rheumatology, Department of Medicine, Solna, Karolinska Institutet, Stockholm, Sweden; ^3^ Division of Pulmonary Medicine, Department of Internal Medicine, Keio University School of Medicine, Shinjuku, Japan; ^4^ Division of Respiratory Medicine, Nagoya University Graduate School of Medicine, Nagoya, Japan; ^5^ Department of Respiratory Medicine, National Tokyo Medical Center, Meguro, Japan; ^6^ Department of Connective Tissue Diseases, National Tokyo Medical Center, Meguro, Japan; ^7^ Department of Dentistry and Oral Surgery, Keio University School of Medicine, Shinjuku, Japan; ^8^ Saitama Medical University, Saitama, Japan

**Keywords:** anti-synthetase syndrome, autoantibody, idiopathic inflammatory myopathy, salivary gland, anti-aminoacyl-tRNA synthetase antibody, anti-Ro52 antibody

## Abstract

Interstitial lung disease is a common complication of anti-synthetase syndrome (ASS), and lymphocytic infiltration is often observed in the lesion. We have recently reported that disease-specific autoantibodies are produced by infiltrating lymphocytes in some autoimmune diseases. Here, we investigate the antigen specificity of B cells in the lung lesions of ASS patients. A total of 177 antibodies were produced from antibody-secreting cells in bronchoalveolar fluid (BALF) of three each of serum anti-Jo-1 and serum anti-EJ antibody–positive patients. Twelve to 30% and 50 to 62% of these antibodies were disease-specific autoantibodies, respectively. These autoantibodies recognized conformational epitopes of the whole self-antigen and had affinity maturations, indicating that self-antigens themselves are the target of humoral immunity. In addition, 100 antibodies were produced from two salivary gland tissues, obtained by chance, of ASS patients. Salivary glands are not generally recognized as lesions of ASS, but unexpectedly, ASS-related autoantibody production was also observed similar to that of BALF. Immunostaining confirmed the presence of ASS-related autoantibody-producing cells in salivary glands. Our results suggest that disease-specific autoantibody production at lesion sites is a common pathogenesis of autoimmune diseases, and that tissue-specific production of autoantibodies can provide insights regarding the distribution of organ manifestations in autoimmune diseases.

## 1 Introduction

The idiopathic inflammatory myopathies (IIMs) are heterogeneous systemic autoimmune diseases characterized by chronic inflammation of the muscles and extra-muscular manifestations. A variety of myositis-specific or myositis-associated autoantibodies have been identified and are associated with specific clinical phenotypes. One of the major groups among them is anti-synthetase syndrome (ASS), characterized by the appearance of anti-aminoacyl-tRNA synthetase antibodies (anti-ARS antibodies) in the serum. Eight types of tRNA synthetase have been identified as antigens, and the common features of ASS are high complication rates of myositis, interstitial lung disease (ILD), skin symptoms (mechanic’s hands), and in some reports, inflammatory arthritis (1–3), although the frequency of manifestations differs slightly depending on the antigens. Another common feature is a relatively good response to steroid treatment but a high relapse rate (4).

There are several hypotheses for the origin of anti-ARS antibodies: molecular mimicry due to the homology of ARS with pathogen-derived antigens such as Epstein-Barr virus; initiation from activation of innate immunity in lungs due to smoking and upper respiratory tract infections; or due to the cleavage of the specially structured his-tRNA by lung granzyme B, which generates a cryptic epitope and is recognized as non-self (5–7). The initial trigger for the development of autoantibodies, however, has not been conclusively established. Meanwhile, it has long been known that serum anti-Jo-1 antibodies appear before the onset of disease, that IgM-type antibodies appear in the early stages, and that anti-Jo-1 antibodies recognize the conformational epitope of the Jo-1 molecule (8). These findings suggest that, after initiation, the native Jo-1 molecule plays a central role in selecting and sustaining anit-Jo-1 antibodies. In particular, lungs are suspected to be a site of anti-Jo-1 antibody production, since anti-Jo-1 antibodies were detected in BALF to the same extent as in serum (9), and similar antibody reactivity was observed in serum and lungs (10). The involvement of T cells is also suspected because anti-Jo-1 antibodies undergo affinity maturation and class switch to mainly IgG1 in the serum, and Jo-1-specific T cells have been reported to be present in the peripheral blood and recently in lung lesions (9, 11, 12). We previously analyzed T cells in bronchoalveolar fluid (BALF) samples from patients with IIMs and found an increase in T peripheral helper cells (Tph)-like PD-1-expressing CD4^+^ T cells, and that the proportion of these cells correlated with the proportion of antibody-producing cells in BALF (13). Pathological reports also showed the infiltration of lymphocytes and plasma cells in lung biopsy tissue from ASS patients (14). These results suggest that B cells may be producing anti-Jo-1 antibody with T cell assistance in the disease lesions of lungs.

While various studies have been conducted on anti-Jo-1 antibody, few studies have been reported on anti-ARS antibodies other than anti-Jo-1, and their detailed profiles remain unknown.

In addition to anti-ARS antibodies, the co-existence of anti-Ro52 antibodies is another characteristic of ASS, with 56 to 71% of cases being positive (15–18). Anti-Ro52 antibodies, along with anti-Ro60 antibodies, have historically been used as markers of Sjögren’s syndrome (SjS) and systemic lupus erythematosus (SLE), but in recent years these two antibodies have been considered to have independent disease associations. SjS and SLE are common in patients with both anti-Ro52 and anti-Ro60 antibodies, while inflammatory myositis and inflammatory rheumatism are common in patients with anti-Ro52 antibodies alone (19, 20). In ASS patients, the presence of anti-Ro52 antibodies is associated with the presence and chronicity of ILD (15, 17). Anti-Ro52 antibodies, however, appear at a certain rate in other autoimmune diseases, and compared to anti-ARS antibodies, it was unclear if they were directly involved in ASS pathogenesis.

We have been focusing on the antibodies produced by infiltrating lymphocytes at the lesion site. A large number of monoclonal antibodies (mAbs) was generated from salivary gland tissue from SjS (21) and BALF samples from rheumatoid arthritis (RA)-, SjS-, and mixed connective tissue disease (MCTD)-associated ILD (22) in previous studies. By creating a panel of monoclonal antibodies derived from disease lesions, the epitopes of each antibody and the effects of somatic hypermutations (SHMs) were examined at single-cell-level resolution, and it was found that disease-specific autoantibodies were produced in approximately one-third of antibody-producing cells in the salivary glands and in 10 to 20% of them in BALF (21, 22). These results suggest that autoantibodies may be commonly produced by infiltrating lymphocytes in systemic autoimmune diseases.

In this study, we directly investigated what antibodies are produced in the lung lesions of serum anti-Jo-1 and also anti-EJ antibody-positive ASS patients, by reconstructing the antibodies produced at the lesion site *in vitro*. Furthermore, we obtained by chance the lip biopsy samples from ASS patients, and report the unexpected results that ASS-associated autoantibodies are produced in the salivary glands, which are not generally recognized as lesions of ASS.

## 2 Materials and methods

### 2.1 Clinical samples

BALF, serum, and lip biopsy samples from patients were collected at Keio University Hospital and National Tokyo Medical Center from June 2016 to September 2022. We enrolled seven patients who met Conners criteria for the ASS (23). Six of them (Pt1–6) underwent bronchoscopy to examine CD4/8 ratio and to rule out other diseases such as infection in patients suspected of having ASS, whose respiratory status was not severely compromised. BALF was used for clinical examination for diagnostic purposes, and excess samples were used for research. Two of them (Pt6, 7) underwent salivary gland biopsy for the diagnosis of SjS, and excess samples were used for research. We also evaluated patients with the 2017 European League Against Rheumatism/American College of Rheumatology classification criteria for IIM (24) and 2016 American College of Rheumatology/European League against Rheumatism Classification Criteria for Primary SjS (25) in order to characterize them. The cohort in the current study did not include the same patients as in the previous study (21, 22). The paired BALF and serum samples were collected on the same day or within a week and without change in treatment, and the lip biopsy sample of Pt 6 was collected on the day following the BALF sample collection without change in treatment. The following parameters were collected from medical charts: anti-nuclear antibody (ANA); serum autoantibodies (measured by ELISA, MBL, Japan), and rheumatoid factor (RF, measured by a latex agglutination test); and medication history. The extent of ILD were scored as previously described (26).

### 2.2 Sample preparation

BALF samples were collected by aspiration immediately after injection of saline in the most-affected area. Samples were filtered through a 75 µm nylon strainer and centrifuged at 500 rpm for 5 min. The supernatants were further centrifuged at 3000 rpm for 10 min and stored at -80°C. A cell pellet was used for cell sorting, as described later. Serum was collected using a serum-separating agent and centrifugation, and was aliquoted and stored at -80°C until use. Labial salivary gland tissues were prepared as previously described (21) with minor modification. In brief, a part of the tissue was mechanically and enzymatically digested in Dulbecco’s modified Eagle’s medium (DMEM) containing 10 mg/ml collagenase type 2 (Worthington, USA) with vigorous shaking at 37°C in 5% CO_2_ for 20 min. After filtering through a 40 μm cell strainer (Greiner Japan, Japan), a single-cell suspension was used for cell sorting, as described later. Another part of the salivary gland tissue was encapsulated in Tissue-Tek O.C.T. Compound (Sakura Finetek, Japan) and used for immunostaining.

### 2.3 Detection of autoantibodies by antigen-binding beads assay

Detection of autoantibodies were performed by antigen-binding beads assay as previously described (21, 22). Briefly, the *HARS1* (coding Jo-1 antigen), *GARS1* (coding EJ antigen)*, NARS1* (coding KS antigen), *TARS1* (coding PL-7 antigen)*, AARS1* (coding PL-12 antigen)*, IARS1, LARS1, MARS1, QARS1, RARS1, KARS1, DARS1, EPRS1*, and *AIMP1–3* (coding OJ antigen) were cloned into the pcDNA3.4 vector combined with the streptavidin-binding peptide (SBP) tag and green fluorescent protein (GFP) at the N-terminus. Point mutated *TRIM21* vector (D335A mutation to remove its Fc receptor activity with minimal impact on conformation, called Ro52m) with SBP tag and GFP was created previously (21). These vectors were transfected into 293T cells (RIKEN BRC, Japan) using polyethyleneimine (Polysciences, USA), and after two days, the cells were lysed in Tris-buffered saline containing 1% Triton X-100 (TBSTx) with a protease inhibitor cocktail (Fujifilm Wako, Japan). The cell lysates were cleared by centrifugation at 16,000 *g* for 15 min at 4°C, and the supernatant was aliquoted and frozen at -80°C until use. Biotinylated SSA/Ro60 was purchased from DIARECT (Germany). The cell lysate including overexpressed antigens or biotinylated Ro60 was incubated with Dynabeads M-280 Streptavidin (Thermo). After washing, the beads were incubated with serum, BALF, or monoclonal antibody in staining buffer, washed, and stained with allophycocyanin (APC)-conjugated anti-human IgGFc antibody. The reactivity against antigen was measured as the median fluorescence intensity (MFI) of APC using FACSVerse (BD, USA), and data were analyzed using FlowJo software (BD).

### 2.4 Comparison of autoantibodies in BALF and serum

The IgG in BALF and serum was purified using Ab Capcher Mag2 (ProteNova, Japan) according to the manufacturer’s instructions. The concentrations of purified IgG from the BALF and serum were measured using a Human IgG ELISA Quantitation Set (Bethyl Laboratories, USA). The comparison of autoantibody titers between BALF and serum was performed by antigen-binding bead assay after adjusting the IgG concentration to 2 μg/ml. The comparison of universal antibody titers between BALF and serum was performed by in-house ELISA. Tetanus toxoid (Merck, Germany, 5 μg/ml) or tuberculin purified protein derivative (Japan BCG Laboratory, Japan, 5 μg/ml) were coated on a microtiter plate overnight. The plates were washed, blocked with ELISA Blocking Buffer (Bethyl Laboratories) for 30 min, washed again, and reacted with 5 μg/ml of purified IgG for 1 h. The plates were washed, reacted with an HRP-conjugated anti-human IgG antibody (Bethyl Laboratories) for 1 h, and reacted with the TMB Substrate Set (BioLegend, USA) for 30 min. Reactions were stopped with H_2_SO_4_, and the optical density at 450 nm (OD 450) was measured.

Because the IgA and IgM are isotypes that are difficult to purify, the concentrations of IgA and IgM of serum and BALF were measured using Human IgA/IgM ELISA Quantitation Set (Bethyl Laboratories), and diluted to 2 μg/ml each, and then antibody titer was measured by beads assay using APC-conjugated anti-human IgA/IgMFc antibody.

### 2.5 Production of monoclonal antibodies from BALF and salivary gland tissue

The cell pellet from BALF was washed with staining buffer (PBS with 0.5% BSA and 2 mM EDTA) and stained with fluorochrome-conjugated antibodies for 15 min. The antibody-secreting cells (CD19^+^sIgD^-^CD27^+^CD38^high^) were bulk-sorted using a FACSAria III flow cytometer (BD), and then single-cell cDNA libraries were created using Smart-seq2 (27). The salivary gland tissue–derived cell suspension was stained with fluorochrome-conjugated antibodies, and single-cell cDNA libraries were created from antibody-secreting cells (7AAD^-^CD3^-^CD4^-^CD8^-^CD326^-^CD19^+^CD38^high^) as in BALF.

IgH, Igλ, and Igκ gene transcripts were amplified and cloned into the expression vector as previously described (22). Briefly, the first polymerase chain reaction was performed using common forward primers and reverse primers designed to bind constant regions, and variable regions were sequenced. The second polymerase chain reaction was performed using cell-specific primers, and amplicons were inserted into the expression vector. Antibodies were produced using the Expi293 Expression System (Thermo), purified using Ab-Capcher Mag2, and checked for purity and concentration. The status of somatic hypermutation and gene usage was analyzed by IMGT/V-QUEST (http://www.imgt.org/IMGT_vquest/vquest). Based on these data, revertant antibodies were produced from representative autoantibodies using synthesized double-stranded DNA fragments (Genewiz, USA) of the variable region in which all of the somatic mutations reverted to the genomic sequence (21, 28).

### 2.6 Polyreactivity ELISA

For mAbs, the polyreactivity of each antibody was tested as previously described (22). Briefly, lipopolysaccharides (LPS, Sigma-Aldrich, USA, 10 μg/ml), insulin (Thermo, 5 μg/ml), or dsDNA (plasmid, 10 μg/ml) were coated on a microtiter plate overnight. The plates were washed, blocked with ELISA Blocking Buffer for 2 h, washed again, and reacted with 2 μg/ml of mAbs for 2 h. The plates were washed, reacted with an HRP-conjugated anti-human IgG antibody for 1 h, and reacted with the TMB Substrate Set for 10 min. Reactions were stopped with H_2_SO_4_, and the OD 450 was measured. We considered antibody binding to two or more of the three antigens to be polyreactive.

### 2.7 Examining reactivity of the mAbs against autoantigens

The reactivity of created mAbs was examined by antigen-binding bead assay. Autoantigen-binding beads were prepared as described above. The beads were incubated with 2 μg/ml of mAbs, washed, and stained with APC-conjugated anti-human IgGFc antibody. The reactivity against antigen was measured as the MFI of APC. The cutoffs were determined as 75% quantile + 5 × (75% quantile - 25% quantile). We also created vectors expressing fragmented autoantigens (Jo-1: 1–401AA, 401–509AA, 1–215AA, 216–415AA, 416–509AA; EJ: 1–538AA, 538–739AA; Ro52m: 1–253AA and 254–475AA), and examined the reactivity against each fragmented autoantigen.

### 2.8 Detection of autoantibody-producing cells in the salivary gland tissue

Immunostaining using GFP-fusion autoantigen for the detection of autoantibody-producing cells was performed as previously described (21). Briefly, the SBP- and GFP-tagged autoantigens (Jo-1, EJ, Ro52m) were purified from the autoantigen-expressing cell lysates described above using Streptavidin Sepharose High Performance (GE Healthcare Japan, Japan). The purities were checked by CBB staining, and the concentrations were measured by a BCA protein assay (Thermo). The SBP-tagged GFP was used for negative control. Ro60-biotin was pre-mixed with Alexa Fluor 488 (AF488)-conjugated Streptavidin (Thermo).

From OCT-embedded salivary gland tissues, 4 μm sections were prepared using cryostat, fixed in acetone for 10 min, blocked with 5% BSA and 10% goat serum in PBS for 10 min at RT, and incubated with 5 μg/ml of SBP-GFP, SBP-GFP-autoantigen, or Ro60-AF488 with anti-CD138 antibody for 60 min at RT. After washing, the slides were incubated with an Alexa Fluor 594–conjugated anti-mouse IgG antibody (1:500 dilution) for 30 min at room temperature. After washing, the slides were mounted with VECTASHIELD Mounting Medium with DAPI (Vector Laboratories, USA), and observed using an LSM710 and ZEN 2.3 SP1 software.

### 2.9 Western blot

The autoantigen-expressing cell lysates were added to 2x SDS sample buffer (Bio-Rad, USA), boiled at 95°C for 5 min, and electrophoresed by SDS-PAGE using a 12.5% 2D-gel (Gellex International, Japan). The proteins were transferred onto polyvinylidene difluoride (PVDF) membranes using the Trans-Blot Turbo Transfer System (Bio-Rad), and blocked with PVDF Blocking Reagent for Can Get Signal (Toyobo, Japan) for 1 h. The membrane was partitioned using Screener Blotter (Samplatec, Japan), and incubated with 0.05 μg/ml of anti-GFP antibody (positive control) or 1 μg/ml of created mAbs. After washing, the membranes were incubated with an HRP-conjugated anti-mouse or anti-human IgG antibody for 1 h. After extensive washing, signals were visualized using ImmunoStar LD (Fujifilm).

### 2.10 Antibody

The following antibodies were used: anti-CD3 (APC/Cy7, UCHT1), anti-CD4 (PE/Cy7, SK3), anti-CD8 (BV510, RPA-T8), anti-CD19 (FITC, HIB19 or BV421, HIB19), anti-sIgD (BV510, IA6–2), anti-CD27 (APC/Cy7, O323), anti-CD38 (PerCP/Cy5.5, HIT2 or FITC, HIT2), anti-CD138 (purified, MI15), anti-CD326 (PE, 9C4), and anti-GFP (purified, 1GFP63) from BioLegend; InVivoMAb human igG1 isotype control from BioXcell (USA); anti-mouse IgG (HRP) from GE Healthcare (USA); and anti-human IgG (HRP) and anti-human IgG/IgA/IgM-Fc (APC or HRP, goat-F (ab’)2 fragment) from Jackson ImmunoResearch (USA).

### 2.11 Statistics

Comparisons of categorical variables were analyzed using Fisher’s exact test. Comparisons of two and multiple continuous variables were performed by a two-sided Wilcoxon’s test and Steel test, respectively. Comparisons of paired continuous variables were performed by a two-sided Wilcoxon signed-rank test. All statistical analyses were performed with JMP 15 (SAS Institute, USA).

### 2.12 Study approval

This study was approved by the Research Ethics Committee of Keio University Hospital (20130246) and National Tokyo Medical Center (R17–151), and conducted in accordance with the Declaration of Helsinki. Written informed consent was obtained from all participating individuals.

## 3 Results

### 3.1 Comparison of the autoantibody titers between BALF and serum

To investigate whether autoantibodies are produced in the lungs of ASS patients, first we collected paired samples of BALF and serum from three each of serum anti-Jo-1 antibody–positive and serum anti-EJ antibody–positive patients who underwent bronchoscopy for the diagnosis of ILD. The characteristics of the patients used in this study are shown in **Table 1**. Because the concentrations of immunoglobulins and other contaminants in BALF and in serum are quite different, initially, IgG was purified from BALF and serum. After adjusting purified IgG concentrations, we compared the autoantibody titers against disease-specific autoantigens, Jo-1 and EJ, and Ro52, which is thought to be strongly associated with ASS. We also measured the titers against Ro60, because three of six patients were diagnosed with SjS.

**Table 1:** Clinical characteristics of patients for BALF analysis.

Patients ID	Pt1	Pt2	Pt3	Pt4	Pt5	Pt6
Age	70	50	43	38	46	72
Sex	Male	Female	Female	Female	Female	Female
Connors criteria for the ASS	Fulfilled	Fulfilled	Fulfilled	Fulfilled	Fulfilled	Fulfilled
2017 ACR/EULAR criteria for IIM^a^ Score points Classification Subgroup	19.4 Definite DM	13.9 Definite PM	13.4 Definite PM	1.3 - (IPAF)	7.8 Probable DM	9.1 Definite PM
Other autoimmune diseases	–	–	–	–	SjS	SjS
Disease onset	relapse	primary	primary	primary	primary	relapse
Disease duration (months)	1	1	1	2	4	4
Clinical symptoms^b^						
Interstitial lung disease Myositis Skin rash Arthritis	+ - - -	+ + - -	+ + - -	+ - - -	+ + + +	+ + - +
ANA titer	<40	<40	<40	<40	<40	<40
ANA type	–	–	–	cyt	cyt	cyt
Serum anti-ARS antibody titer^c^	35.1	132	101.8	168.7	166.9	186.6
Serum anti-Jo-1 antibody titer^d^	600	19595	19595	138	102	102
Serum anti-EJ antibody titer^d^	105	120	113	11271	26499	31670
Serum anti-Ro60 antibody titer^e^	1.2	NA	NA	6.5	229.3	26.3
Serum anti-SSB antibody titer^e^	<1.0	NA	NA	<1.0	9.1	<1.0
HRCT pattern	OP	NSIP	NSIP	NSIP	NSIP	NSIP
ILD extent (%)	20	23	28	8	12	18
Collected BALF amount (ml)	38	37	48	89	87	60
CD4/8 ratio	1.38	0.37	0.13	0.15	0.16	0.90
Medication	PSL8 mg, CyA50 mg	no	no	no	no	no
Single-cell cDNA production	48	38	48	96	10	8
mAb production	41	33	45	45	6	7

a At the time of diagnosis.

b At the time of sample collection.

c From medical records, measured by enzyme-linked immunosorbent assay.

d Median fluorescence intensity measured by bead assay.

e From medical records, measured by chemiluminescence enzyme immunoassay.

DM, dermatomyositis; PM, polymyositis; ILD, interstitial lung disease; SjS, Sjögren’s syndrome; ANA, anti-nuclear antibody test (cyt, cytoplasmic); NA, not assessed; HRCT, high-resolution computed tomography; OP, organizing pneumonia; UIP, usual interstitial pneumonia; NSIP, nonspecific interstitial pneumonia; PSL, prednisolone; CyA, cyclosporine A; mAb, monoclonal antibody.

**Figure 1:**
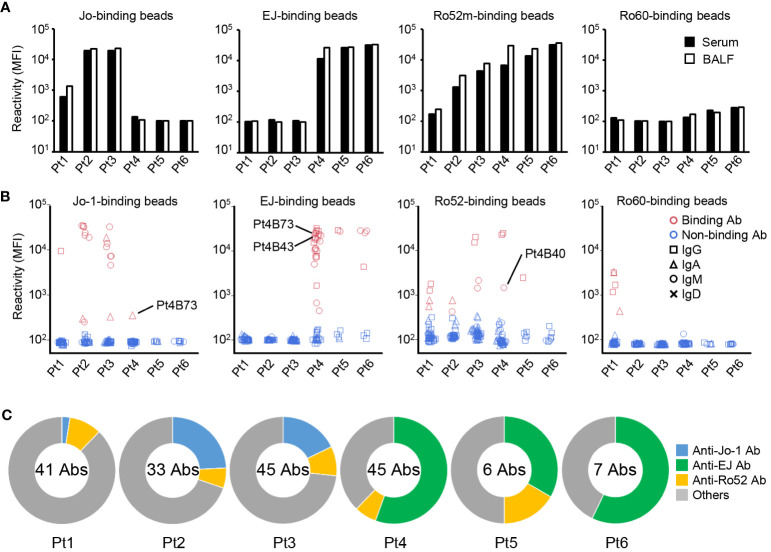
Autoantibody production in the lungs. **(A)** IgG was purified from paired samples of serum and bronchoalveolar fluid (BALF), and the reactivity against Jo-1-, EJ-, Ro52m-, and Ro60-binding beads was measured as median fluorescence intensity (MFI) by antigen-binding bead assay. **(B)** Monoclonal antibodies were generated from antibody-secreting cells in BALF. The reactivity of these antibodies against Jo-1-, EJ-, Ro52m-, and Ro60-binding beads was measured as MFI by bead assay. Antibodies that bound to the beads are shown in red and those that did not are shown in blue. The isotype of the antibodies are shown by shapes. Pt4B73 could bind to both Jo-1 and EJ. Pt4B40 and Pt4B43 are polyreactive antibodies. **(C)** The proportion of ASS-related autoantibodies among the antibodies derived from each patient are shown. The center of the graph indicates the number of antibodies produced from each patient.

As shown in **Figure 1A**, the titer of the anti-Jo-1 antibody of BALF from serum anti-Jo-1 antibody–positive patients was slightly higher (1.12–2.29 times) than serum; similarly, the titer of the anti-EJ antibody of BALF from serum anti-EJ antibody–positive patients was slightly higher (1.03–2.29 times) than serum. The titer of anti-Ro52 antibodies were on average 2.13 times higher in BALF than in serum. It was assumed that there would be leakage of antibodies from the serum into BALF. To demonstrate that the differences of autoantibody titers were not due to a technical variation in the concentration of the purified antibody, antibody titers against the control antigen were also measured. As shown in **Supplementary Figure 1**, serum had similar or higher titers than BALF against tetanus toxoid or tuberculosis. Although the number of samples is small and the difference in antibody titers is not large, these results indicated the possibility that some of the autoantibodies may be produced in the lung lesions. Serum anti-Ro60 antibody titers were not high in all patients, and BALF was similar.

### 3.2 Reactivity of monoclonal antibodies produced from BALF ASCs

Next, we produced monoclonal antibodies from ASCs in BALF to directly examine the production of autoantibodies in the lungs. From six BALF samples, 177 monoclonal antibodies were produced. We first checked the polyreactivity of these antibodies to exclude the effects of nonspecific binding, and found three polyreactive antibodies among them (Pt3B36, Pt4B40, and Pt4B43, **Supplementary Figure 2A**). Then, the reactivity of these antibodies were examined by antigen-binding bead assay using Jo-1-, EJ-, Ro52m-, and Ro60-binding beads (**Figure 1B**). Some antibodies produced from serum anti-Jo-1 antibody–positive patients (Pt1–3) reacted with Jo-1, and some antibodies produced from serum anti-EJ antibody–positive patients (Pt4–6) reacted with EJ. The proportion of anti-Jo-1 antibody was low in antibodies derived from Pt 1, who was a relapse case and was under immunosuppressive treatment, and whose serum anti-Jo-1 antibody titer was low. Anti-Ro52 antibodies, which are frequently observed in the sera of ASS patients, were also cloned from five of six patients. Curiously, four anti-Ro60 antibodies were cloned from Pt1, whose serum anti-Ro60 antibody was negative.

To verify whether anti-ARS antibodies, which are mutually exclusive at the serum level (29), are not cross-reactive with other ARS molecules even at the monoclonal level, we examined their reactivity against other ARS molecules (KS, PL-7, PL-12, and OJ). As shown in **Supplementary Figure 2B**, most of anti-Jo-1, anti-EJ, and anti-Ro52 antibodies could not bind other ARS molecules. Four antibodies reacted weakly to KS and PL-7, among which Pt4B73 was the antibody that bound strongly to EJ and weakly to Jo-1, and Pt4B40 was a polyreactive antibody. The proportion of autoantibodies in each patient is shown in **Figure 1C**. Compared to anti-Jo-1 antibodies, anti-EJ antibodies were identified very frequently, in approximately one-half of the cloned antibodies. To show that the reactivity of these antibodies to ARS molecules is specific to ASS, we confirmed that antibodies previously generated from BALF of RA-ILD (22) could not bind to Jo-1 and EJ (**Supplementary Figure 2C**). These results directly demonstrate that antibodies specific for ASS are produced in the lung lesions of ASS.

The isotypes of the autoantibodies obtained were analyzed later, but there were more IgM autoantibody than expected. Therefore, we checked BALF and serum for the presence of IgM and IgA autoantibodies. Similar to IgG antibodies, IgA and IgM anti-Jo-1 antibodies were detected in serum anti-Jo-1 antibody–positive patients (Pt1–3), and IgA and IgM anti-EJ antibodies in serum anti-EJ antibody–positive patients (Pt4–6) (**Supplementary Figure 2D**). The titer of IgA autoantibodies were higher in BALF than serum, as in IgG antibodies, whereas those of IgM autoantibodies were variable. The autoantibody titers were higher in IgM antibodies than in IgA antibodies, consistent with the previous studies (11, 30). In contrast, anti-Ro52 antibody titers of IgA and IgM were not high, and IgG was considered to be predominant.

### 3.3 Reactivity of monoclonal antibodies produced from salivary glands

Pt6 had xerostomia and underwent salivary gland biopsy for the diagnosis of SjS. Histological examination showed foci in the salivary glands, and together with the serum anti-Ro60 antibody, Pt6 could be classified as SjS. At this time, we collected the excess salivary gland tissue for research. In addition, another salivary gland sample from a serum anti-Jo-1 antibody–positive ASS patient, who could also be classified as SjS, was collected. Patient background is shown in **Table 2**.

**Table 2:** Clinical characteristics of patients for salivary glands analysis.

Patients ID	Pt7	Pt6
Age	70	72
Sex	Female	Female
Connors criteria for the ASS	Fulfilled	Fulfilled
2017 ACR/EULAR criteria for IIM^a^ Score points Classification Subgroup	12.1 Definite DM	9.1 Definite PM
2016 ACR/EULAR criteria for SjS	Fulfilled	Fulfilled
Other autoimmune diseases	SSc	–
Disease duration (months)	60	4
Clinical symptoms^b^		
Interstitial lung disease Myositis Skin rash Arthritis	+ + + -	+ + - -
ANA titer	320	<40
ANA type	s	cyt
Serum anti-ARS antibody titer^c^	176.8	186.6
Serum anti-Jo-1 antibody titer^d^	5467	102
Serum anti-EJ antibody titer^d^	75	31670
Serum anti-Ro60 antibody titer^e^	<1.0	26.3
Serum anti-SSB antibody titer^e^	<1.0	<1.0
Medication	no	no
Gum test (ml/10 min)	1.3	3.8
Schirmer test, right, left (mm/5 min)	2, 1	2, 4
SPK	positive	negative
Focus score	>1	>1
Single cell cDNA production	80	32
mAb production	77	23

a At the time of diagnosis.

b At the time of sample collection.

c From medical records, measured by enzyme-linked immunosorbent assay.

d Median fluorescence intensity measured by bead assay.

e From medical records, measured by chemiluminescence enzyme immunoassay.

DM, dermatomyositis; SSc, systemic sclerosis; SjS, Sjögren’s syndrome; ANA, anti-nuclear antibody test (s, speckled; cyt, cytoplasmic); NA, not assessed; SPK, superficial punctate keratopathy; mAb, monoclonal antibody.

**Figure 2:**
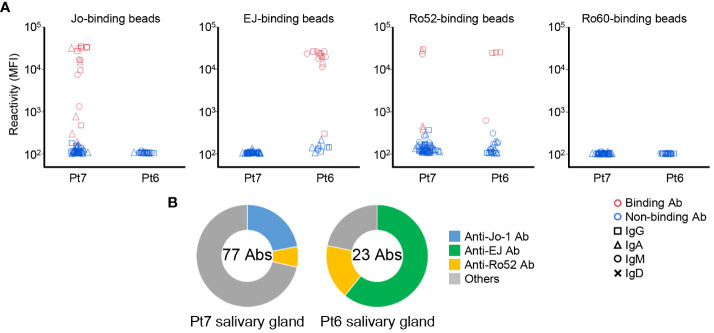
Autoantibody production in the salivary glands. **(A)** Monoclonal antibodies were generated from antibody-secreting cells in salivary gland tissues. The reactivity of these antibodies against Jo-1-, EJ-, Ro52m-, and Ro60-binding beads were measured as MFI. Antibodies that bound to the beads are shown in red and those that did not are shown in blue. The isotype of the antibodies are shown by shapes. **(B)** The proportion of ASS-related autoantibodies among the antibodies derived from each patient are shown. The center of the graph indicates the number of antibodies produced from each patient.

In the previous study (21), we showed that anti-Ro60 antibodies could be found from the salivary glands of serum anti-Ro60 antibody–positive patients. In the same way, we produced monoclonal antibodies from ASCs infiltrated in salivary glands, and examined the reactivity against Ro60 and, to be prudent, also against Jo-1, EJ, and Ro52 by antigen-binding bead assay. Surprisingly, the antibodies produced from the salivary glands of serum anti-Jo-1 antibody–positive patients (Pt7) reacted with Jo-1 and Ro52, and the antibodies produced from the salivary glands of serum anti-EJ antibody–positive patients (Pt6) reacted with EJ and Ro52, the same as for BALF (**Figure 2A**). In contrast, no antibodies reacted with Ro60, even though Pt6 had serum anti-Ro60 antibody. The proportions of autoantibodies in the salivary glands are shown in **Figure 2B** and were similar to those of BALF, suggesting that a similar immune response occurs in the salivary glands as in the lungs. We also checked the polyreactivity of these antibodies, but no antibody was considered to be polyreactive (**Supplementary Figure 3**).

### 3.4 Evaluation of autoantibody production in salivary gland tissue by immunostaining

**Figure 3:**
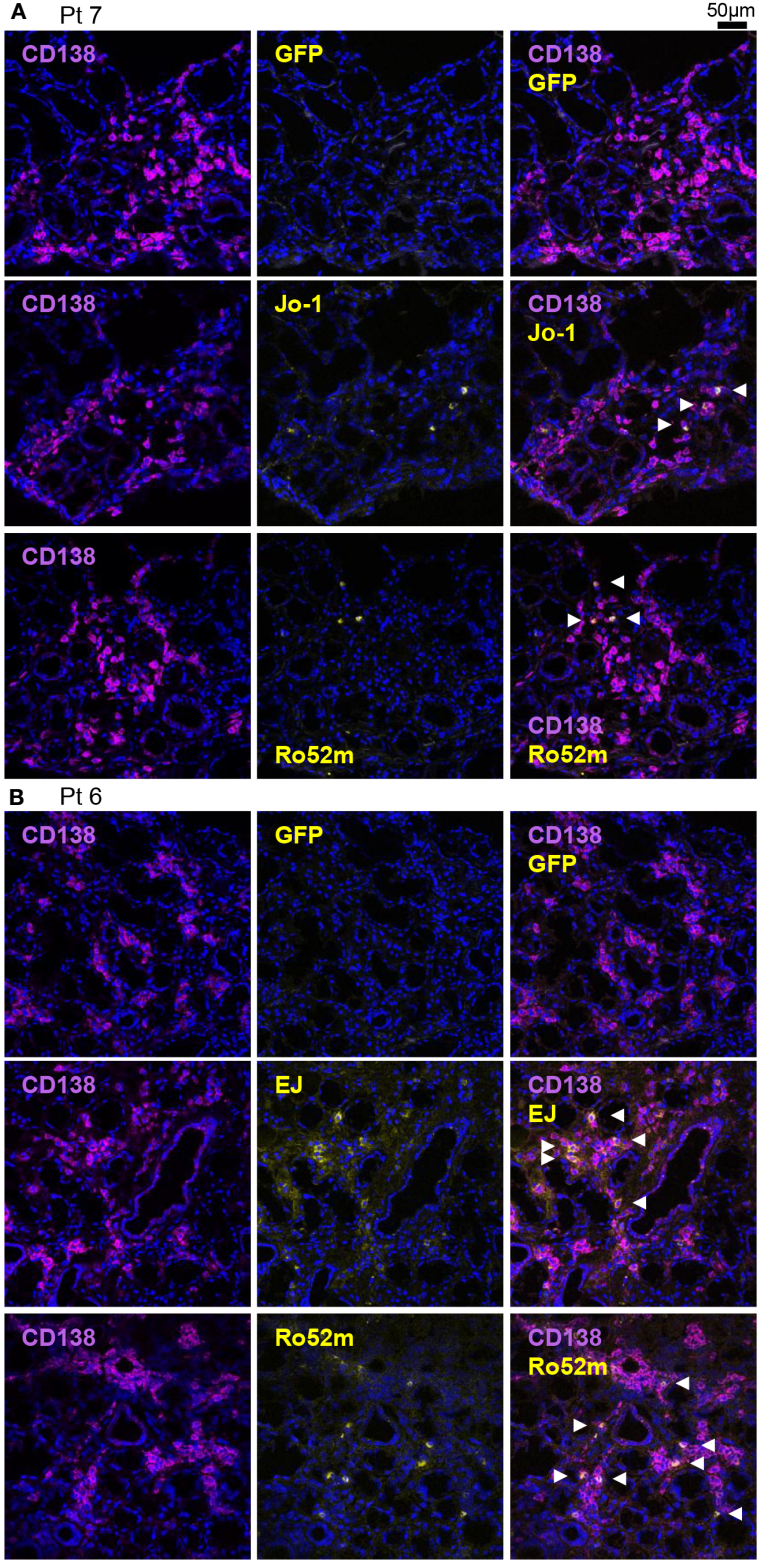
Detection of autoantibody-producing cells by immunofluorescence using autoantigens. Fresh-frozen sections of salivary glands were stained with purified green fluorescent protein (GFP) or GFP-autoantigen fusion proteins, anti-CD138 antibody (a marker of the antibody-producing cells), and DAPI. Autoantibody-producing cells are defined as GFP-autoantigen-positive and anti-CD138 antibody–positive cells and are indicated by white arrowheads. Representative single marker and overlay images of the salivary gland **(A)** from patient 7 (serum anti-Jo-1 antibody–positive) and **(B)** from patient 6 (serum anti-EJ antibody–positive) are shown. Scale bar indicates 50 μm.

To confirm the production of ASS-related autoantibodies in salivary glands in another experimental procedure, immunostaining was performed in the above two cases and in serum anti-Ro60 antibody-positive typical SjS patients. Patient background is shown in **Supplementary Table 1**. GFP-fused autoantigens were prepared and then reacted with the tissue slides to determine whether the autoantigens bind to antibodies in the cytoplasm of ASCs in salivary gland tissues. We confirmed that anti-Jo-1 and anti-Ro52 antibody–producing cells were indeed present in the salivary glands of Pt7 (**Figure 3A**), and that anti-EJ and anti-Ro52 antibodies were indeed present in the salivary glands of Pt6 (**Figure 3B**). Consistent with the reactivity of monoclonal antibodies, no anti-Ro60 antibody–producing cells were found in either tissue (**Supplementary Figure 4A**). In contrast, anti-Ro60 and anti-Ro52 antibody–producing cells, but not anti-Jo-1 and anti-EJ antibody–producing cells, were present in salivary glands from serum anti-Ro60 antibody–positive typical SjS patients (**Supplementary Figure 4B**). Although the number of specimens was very small, these results confirm that ASS-related autoantibody production could occur in the salivary glands as well as in the lungs.

### 3.5 Distribution of epitopes of monoclonal autoantibodies

**Figure 4:**
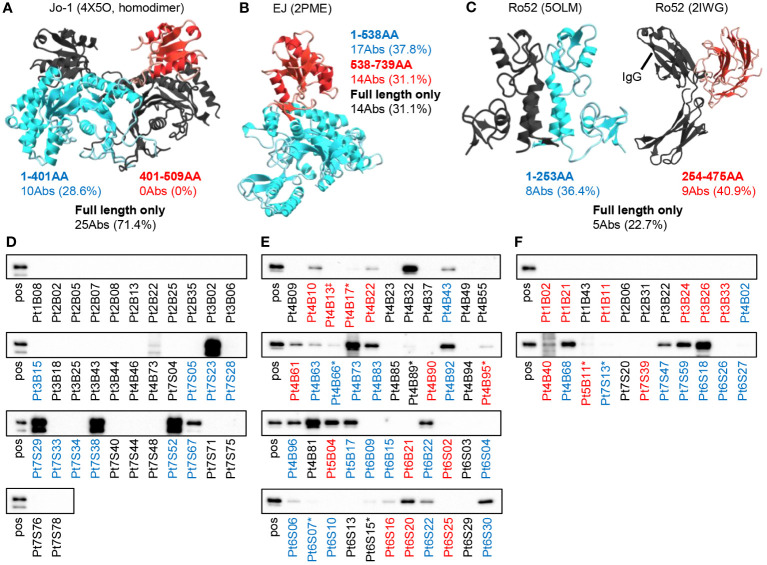
Binding mode of autoantibodies against autoantigen. The reactivities of autoantibodies to full-length or fragmented autoantigen-binding beads were examined. The number of antibodies that can recognize each fragment (red or blue) or only the full-length antigen (black) is shown. The figure shows the three-dimensional structures of **(A)** Jo-1 (4X5O), **(B)** EJ (2PME), and **(C)** Ro52 (5OLM and 2IWG) reported in PDB, colored by the fragment. Note that the reported structure does not include the positions of some amino acids. Since the full-length structure of Ro52 is not known, the two structural analyses were cited separately. **(D–F)** We performed western blotting to determine whether autoantibodies could recognize GFP-fused denatured antigens ((D) Jo-1, **(E)** EJ, and **(F)** Ro52m) on the membrane. “pos” indicates blotted with anti-GFP antibody, and other lane names indicate the name of the antibody blotted. The antibody name indicates the patient number of origin, whether BALF **(B)** or salivary gland (S) derived, and the cell number of origin. The colors of the antibody names were matched to the results in **(A–C)**. * indicates very weak binding that is rather difficult to see in the figure. ‡Since the sequences of Pt4B13 and Pt4B14 are identical, only Pt4B13 was examined.

Next, we examined which parts of the antigen the antibodies recognize. We expressed Jo-1, EJ, and Ro52 antigens as two separate parts and examined the binding of the antibodies by bead assay. **Figures 4A–C** shows how the antigens were divided and how many antibodies bound to each fragmented antigen. Some anti-EJ and anti-Ro52 antibodies could bind to fragmented antigens and some could bind only to full-length antigens, whereas a small number of anti-Jo-1 antibodies could bind to fragmented antigens. We therefore further examined the Jo-1 antigen by dividing it into three fragments at different positions, but even so, few antibodies recognized fragmented antigens (**Supplementary Figure 5**), indicating that conformation is important for Jo-1 recognition, consistent with a previous serum-based study (8).

To investigate whether the antibodies recognize linear epitopes by a different approach, we performed western blotting. As shown in **Figures 4D–F**, a certain proportion of the anti-EJ and anti-Ro52 antibodies recognized linear epitopes (55.6% and 31.8%, respectively), whereas anti-Jo-1 antibodies did not recognize as much (17.1%). Antibodies colored black in **Figures 4D–F**, which could only recognize full-length antigens in the bead assay, exhibited low reactivity in Western blotting, consistent with antibodies assumed to recognize conformational epitopes. These results indicate that autoantibodies are produced against various sites of the autoantigens, including the three-dimensional structure.

### 3.6 Antigen-driven selection of autoantibodies

**Figure 5:**
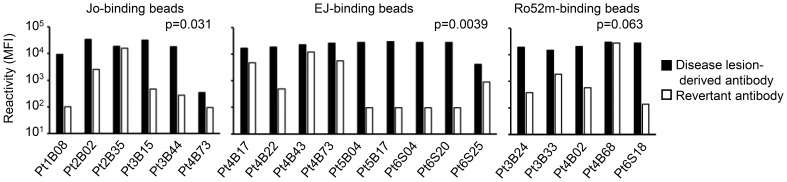
Affinity maturation of the autoantibodies. Representative revertant antibodies were produced by reverting the somatic hypermutations of the disease lesion–derived antibodies to the genomic sequence. The reactivities of disease lesion–derived antibodies and revertant antibodies against corresponding antigens were compared. Disease lesion–derived Pt4B73 could bind to both Jo-1 and EJ. Wilcoxon signed-rank test.

To investigate whether these antibodies are actually produced in an antigen-driven manner at the lesion site, we next focused on somatic hypermutations (SHMs). We generated revertant antibodies from representative autoantibodies in which SHMs were reverted to the genomic sequence, and compared the reactivity of the disease lesion–derived autoantibodies and their revertant antibodies. As shown in **Figure 5**, most of the revertant antibodies showed decreased or lost reactivity against corresponding antigens, indicating that the autoantibodies are selected and refined against autoantigens by accumulating SHMs.

### 3.7 Sequence analysis of the created antibodies

**Figure 6:**
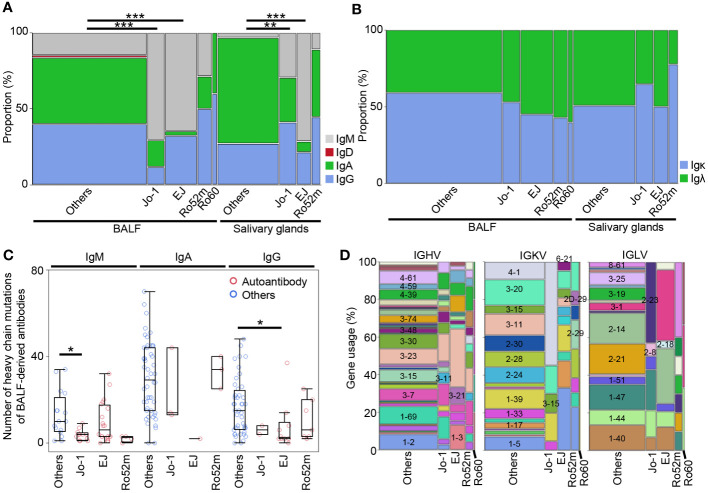
The characteristics of autoantibodies. **(A)** H-chain and **(B)** L-chain isotypes for each corresponding autoantigen of BALF or salivary gland-derived antibodies were compared with other antibodies by Fisher’s exact test with Bonferroni correction. **p < 0.01, ***p < 0.001. Because there were only two IgDs, we excluded them from subsequent analysis. **(C)** The number of the somatic hypermutations of heavy chain of the antibodies from BALF samples were compared between autoantibodies and other antibodies by Steel test. *p < 0.05. **(D)** The gene usage of the antibodies are shown.

To examine the sequence characteristics of autoantibodies, we analyzed the isotype, the number of SHMs, and gene usage. **Figure 6A** shows the proportion of heavy- and light-chain isotype by corresponding antigens. Similar to both BALF- and salivary gland–derived antibodies, the proportion of IgM of anti-Jo-1 antibodies and anti-EJ antibodies was significantly higher than antibodies with unknown specificity. The proportion of IgM of anti-Ro52 antibodies was not high, consistent with the autoantibody titer in BALF and serum as shown in **Supplementary Figure 2C**. No significant differences were observed in light chain (**Figure 6B**).

Next, we compared the number of SHMs in the variable region of the antibodies by corresponding antigens. Because class-switched antibodies have more mutations than IgM in general, the comparisons were performed within each isotype. In IgM and IgG, where many autoantibodies were cloned, the overall number of SHMs tended to be lower for autoantibodies than for other antibodies, and significantly lower for heavy and light chain of IgM-type anti-Jo-1 antibody and for heavy chain of IgG-type anti-EJ antibody (**Figure 6C**, **Supplementary Figures 6A–C**). For salivary gland-derived antibodies, only the IgG-type anti-Ro52 antibody had fewer SHMs, however, due to the small sample size, this is a reference value (**Supplementary Figures 6D–G**).

We previously produced monoclonal antibodies from BALF samples of patients with RA-, SjS-, and MCTD-associated ILD (22). Compared to that study, the autoantibodies in this study were significantly more IgM, and the number of SHMs for both IgM and IgG types of autoantibodies was significantly lower (**Supplementary Figures 7A, B**), which were considered characteristic of the autoantibodies in this study.

Finally, the gene usage of IGHV, IGKV, and IGLV genes were checked (**Figure 6D**). The gene usage of autoantibodies was particularly biased toward the light chain, with IGKV4–1 and IGLV2–23 being most common for anti-Jo-1 antibodies, IGKV1–5, IGLV3–1, and IGLV2–14 for anti-EJ antibodies, and IGKV1–5 and IGRV8–61 for anti-Ro52 antibodies. In contrast, IGHVs were diverse, with anti-EJ antibodies having more IGHV3–23 and IGHV1–3, but not as much bias as light chain. There are few papers on gene usage of anti-ARS antibodies, and one recent paper reported that IGHV3–23/IGKV4–1 is the most frequent in anti-Jo-1 antibody-producing peripheral B cells (31), which is partly consistent with our results.

These results indicate that autoantibodies of diverse repertoires are produced and mature against various epitopes of the ARS molecule in the lung lesions of the ASS patients.

## 4 Discussion

In this study, we directly demonstrated that two types of anti-ARS antibodies, anti-Jo-1 and anti-EJ antibodies, and anti-Ro52 antibodies, which are frequently positive in ASS, are produced in the lung lesions in an antigen-driven manner. The majority of anti-Jo-1 antibodies identified in this study recognized the conformational epitope of Jo-1, similar to what has been reported in previous serum studies (8). In addition, the majority of anti-EJ and anti-Ro52 antibodies recognized fragmented antigens and denatured antigens in western blotting, similar to previous reports that those antibodies in serum recognize *Escherichia coli*–derived antigens and synthetic peptides without conformations (32, 33). The characteristics of autoantibodies produced at the disease lesion in this study are similar to those of salivary glands in SjS and those of BALF samples from RA-, SjS-, and MCTD-ILD, which we have reported recently (21, 22), reinforcing our hypothesis that disease-specific autoantibodies are commonly produced by infiltrating lymphocytes in systemic autoimmune diseases.

In addition, by examining antibody reactivity at the single-cell level, we identified several new findings that have not been observed in previous serum-based studies. First, most of the anti-ARS antibodies produced in the lung lesion were of IgM, and there were fewer SHMs in IgM- and IgG-autoantibodies than in the other antibodies. This may suggest that the immune response against Jo-1, and EJ has recently begun. The lung lesions associated with ASS are progressive and are often noticed early, and in fact, the disease duration of the cases in this study was 1 to 4 months, which is much shorter than the average of more than 10 years in previous studies examining BALF-derived antibodies in RA, MCTD, and SjS (22). In contrast, anti-Ro52 antibodies did not show a higher proportion of IgM compared to anti-ARS antibodies. Anti-Ro52 antibodies are known to appear widely in autoimmune diseases other than ASS, suggesting that different mechanisms of production may exist.

Second, anti-EJ antibodies were found in the highest proportion, about half of the antibodies produced, in all three BALF cases and one salivary gland case in common, compared to the anti-Jo1 antibodies examined in the present study and to the anti-citrullinated protein antibodies and anti-RNP antibodies examined in previous studies (21, 22). Because the sample size is small, further case accumulation is needed.

Most surprising was the production of ASS-related autoantibodies in the salivary glands. In addition to examining the reactivity of antibodies produced from salivary glands, we confirmed this finding with another approach by staining the tissue with GFP-fused autoantigens. The two cases analyzed in this study both had subjective symptoms of dry mouth, showed decreased saliva and tear fluid, and had foci on salivary glands; one of them was positive for serum anti-Ro60 antibody, resulting in satisfying the classification criteria for SjS. Therefore, generally they would have been diagnosed with secondary SjS complicated by ASS. The antibodies produced in the salivary glands, however, contained anti-Jo-1, anti-EJ, and anti-Ro52 antibodies in similar proportions to BALF, indicating that similar autoimmune reactions occur in the salivary glands as in the lung lesions.

In a recent study of more than 500 myositis patients with myositis-associated autoantibodies, classification by autoantibodies was more effective in building homogenous subgroups than 2017 classification criteria by IIM criteria (34). Similarly, we previously showed that anti-centromere antibody (ACA) found in the sera and salivary glands of SjS and those found in the sera of systemic sclerosis (SSc) and primary biliary cholangitis (PBC) are identical, with the same reactivity (21), and the clinical manifestations such as Raynaud’s symptoms and sclerodactyly are also common in serum ACA-positive patients regardless of their diseases (35). In other words, although SjS is a disease in which a variety of antibodies appear, classification by autoantibody may be useful in grouping patients with similar disease phenotypes. With the same concept, and based on similar autoimmune reactions observed in the salivary glands and lungs, the salivary gland lesions found in this study may be better classified as ASS-associated lesions. However, this is a preliminarily report, as only two patients in this study had salivary gland lesions producing anti-ARS antibodies. Future evaluation of salivary gland lesions in ASS patients, including those with or without xerostomia symptoms, is warranted.

As noted above, a limitation of this study is the small number of cases. Bronchoscopy for the diagnosis of ASS may be omitted if the patient is elderly, in poor respiratory condition, or if the patient refuses the procedure because of its invasiveness. In addition to the rarity of the disease itself, ASS has multiple corresponding antigens, making the collection of BALF samples with specific autoantibodies very difficult. In fact, there was only one paper that examined anti-Jo-1 antibodies at the monoclonal level using peripheral blood (31), and no other report examined in detail at the lesion site. Fewer reports are available on antibodies other than Jo-1, and this report is the first study on patient-derived anti-EJ antibodies in detail. Although the number of cases was small, by producing and examining a large number of antibodies directly from the lesion sites, we were able to clearly show that the disease-specific autoantibodies were produced in the lung lesions. For salivary gland samples, there were only two cases, because this tissue has not been considered as an affected organ in ASS. Future case series are desirable to determine the extent to which salivary gland lesions producing ASS antibodies are present.

Studies of antigen specificity of the antibodies produced at the lesion site, as in this study, have only just begun with the development of single-cell analysis techniques, and it will be necessary to examine anti-ARS antibodies other than anti-Jo-1 and anti-EJ antibodies, and whether autoantibody production occurs in affected organs other than the lung and salivary glands. Antibodies recognize the structure of their targets; the targets of anti-ARS antibodies in ASS are functionally similar but structurally different, and the presence of anti-ARS antibody is mutually exclusive (29). The fact that ASS patients with autoantibodies to different antigens can develop common clinical symptoms in the muscles, lungs, etc., is of great scientific interest. The findings of this study highlight the importance of analyzing antigen specificity at the single-cell level in disease lesions to elucidate the immunopathology involved in systemic autoimmune diseases, which cannot be achieved by serum studies.

## Data availability statement

The original contributions presented in the study are included in the article/**Supplementary Material**. Further inquiries can be directed to the corresponding author.

## Ethics statement

The studies involving humans were approved by the Research Ethics Committee of Keio University Hospital and National Tokyo Medical Center. The studies were conducted in accordance with the local legislation and institutional requirements. The participants provided their written informed consent to participate in this study.

## Author contributions

MT: Writing – review & editing, Writing – original draft, Methodology, Investigation, Funding acquisition, Conceptualization. KS: Writing – review & editing, Conceptualization. MN: Writing – review & editing, Investigation, Data curation. HK: Writing – review & editing, Resources, Investigation. MI: Writing – review & editing, Resources, Investigation. YO: Writing – review & editing, Resources, Investigation. HO: Writing – review & editing, Supervision, Funding acquisition. SU: Writing – review & editing, Resources. KT: Writing – review & editing, Resources. TT: Writing – review & editing, Supervision, Funding acquisition.
