# Supplementary material for: Disease-specific autoantibody production in the lungs and salivary glands of anti-synthetase syndrome
Source: Front Immunol. 2024 Jun 13;15:1265792. doi: 10.3389/fimmu.2024.1265792 (PMC11208671; doi:10.3389/fimmu.2024.1265792)
Supplement: Supplementary file 1 [file DataSheet_1.pdf]

Supplementary Material

Disease-specific autoantibody production in the lungs and salivary glands of anti-synthetase syndrome

Masaru Takeshita[*], Katsuya Suzuki, Maho Nakazawa, Hirofumi Kamata, Makoto Ishii, Yoshitaka Oyamada, Hisaji Oshima, Satoshi Usuda, Kazuyuki Tsunoda, Tsutomu Takeuchi

*** Correspondence:** Corresponding Author: takeshita@a5.keio.jp

1 Supplementary Figures and Tables

1.1 Supplementary Figures

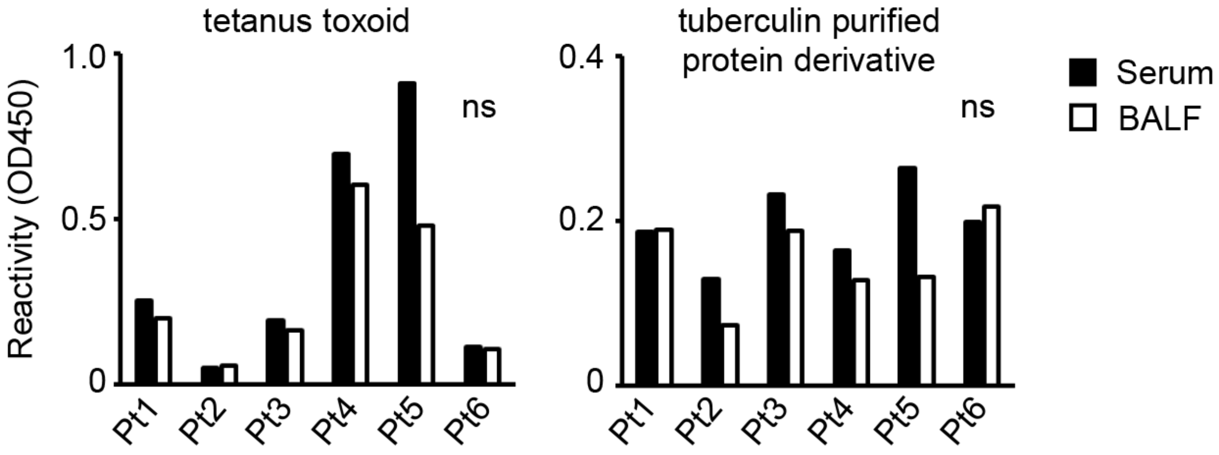

Supplementary Figure 1. Comparison of serum and BALF antibody titers to control antigens

IgG was purified from paired samples of serum and BALF, and the reactivity against tetanus toxoid and tuberculin purified protein derivative was measured by ELISA at 5 μg/ml of purified IgG. Wilcoxon signed-rank test.

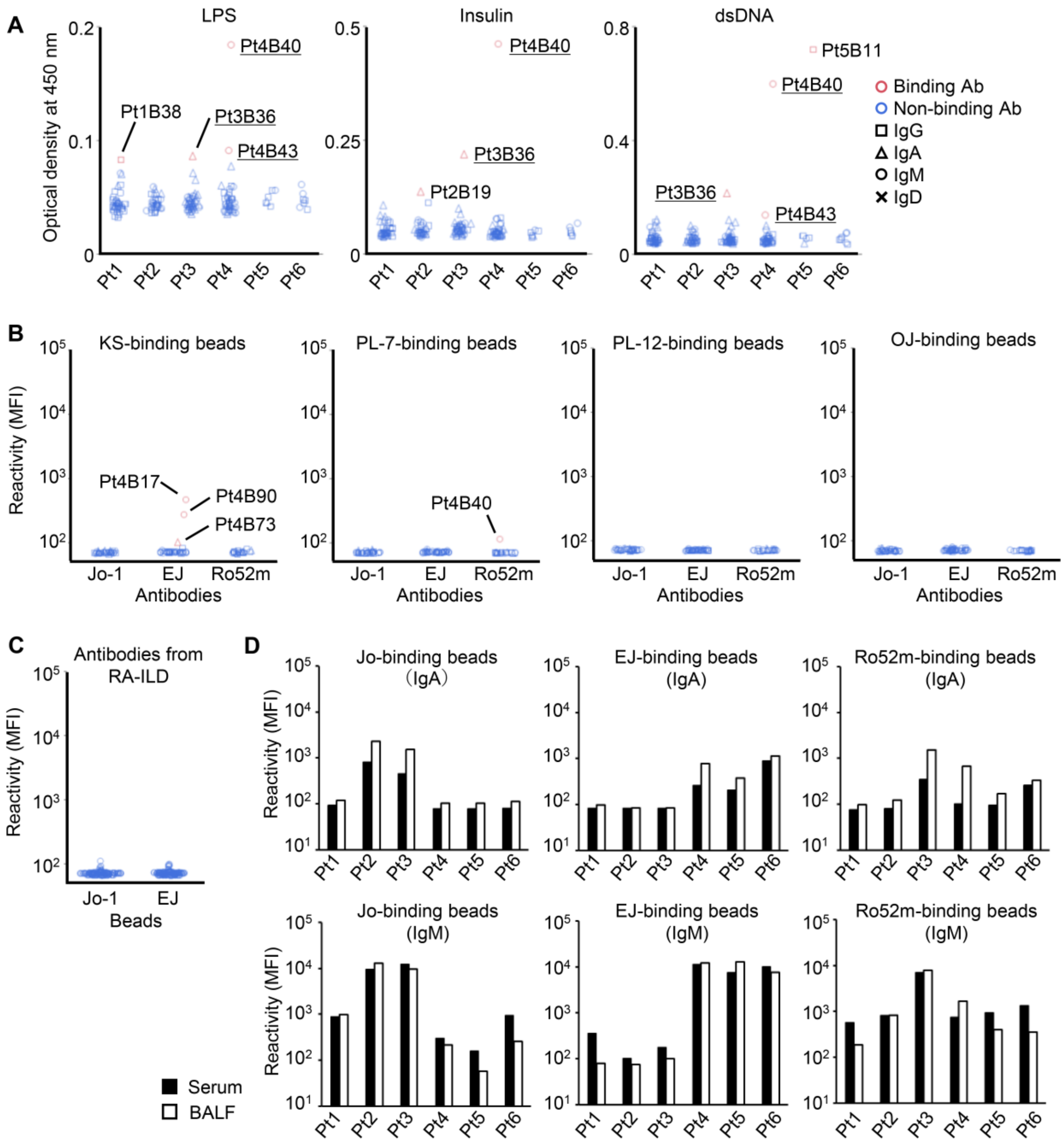

Supplementary Figure 2. The reactivity of antibodies produced from lung lesions

(A) The polyreactivity of BALF-derived antibodies was examined by enzyme-linked immunosorbent assay. The optical density of each antibody against lipopolysaccharide (LPS), insulin, and dsDNA are shown. The antibodies that can bind to two or more antigens are defined as polyreactive antibodies and are underlined. (B) The reactivity of anti-Jo-1, anti-EJ, and anti-Ro52 antibodies against KS-, PL-7-, PL-12-, and OJ-binding beads were measured as MFI. The isotype of the antibodies are shown by shapes. (C) The reactivity of 123 antibodies produced from RA-ILD against Jo-1- and EJ-binding beads were measured as MFI. Antibodies that bound to the antigen are shown in red with the name of the antibody, and those that did not are shown in blue. (D) After the concentrations of IgA and IgM of serum and BALF are adjusted to 2 μg/ml, the reactivity against Jo-1-, EJ-, Ro52m-binding beads was measured as MFI by antigen-binding bead assay.

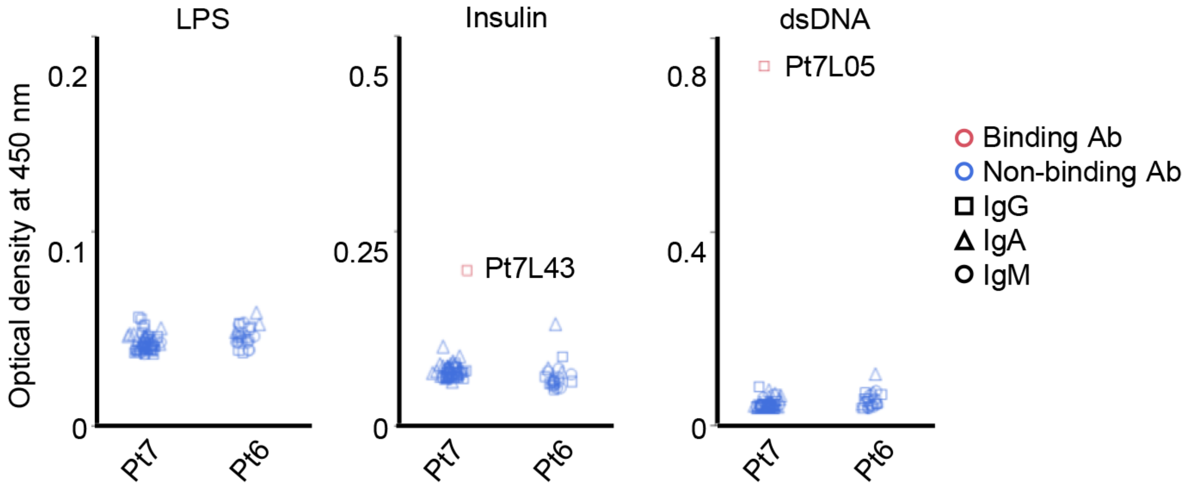

Supplementary Figure 3. Detection of polyreactive antibodies

The polyreactivity of salivary gland–derived antibodies was examined by enzyme-linked immunosorbent assay. The optical density of each antibody against lipopolysaccharide (LPS), insulin, and dsDNA are shown. Antibodies that bound to the antigen are shown in red with the name of the antibody, and those that did not are shown in blue. The isotype of the antibodies are shown by shapes.

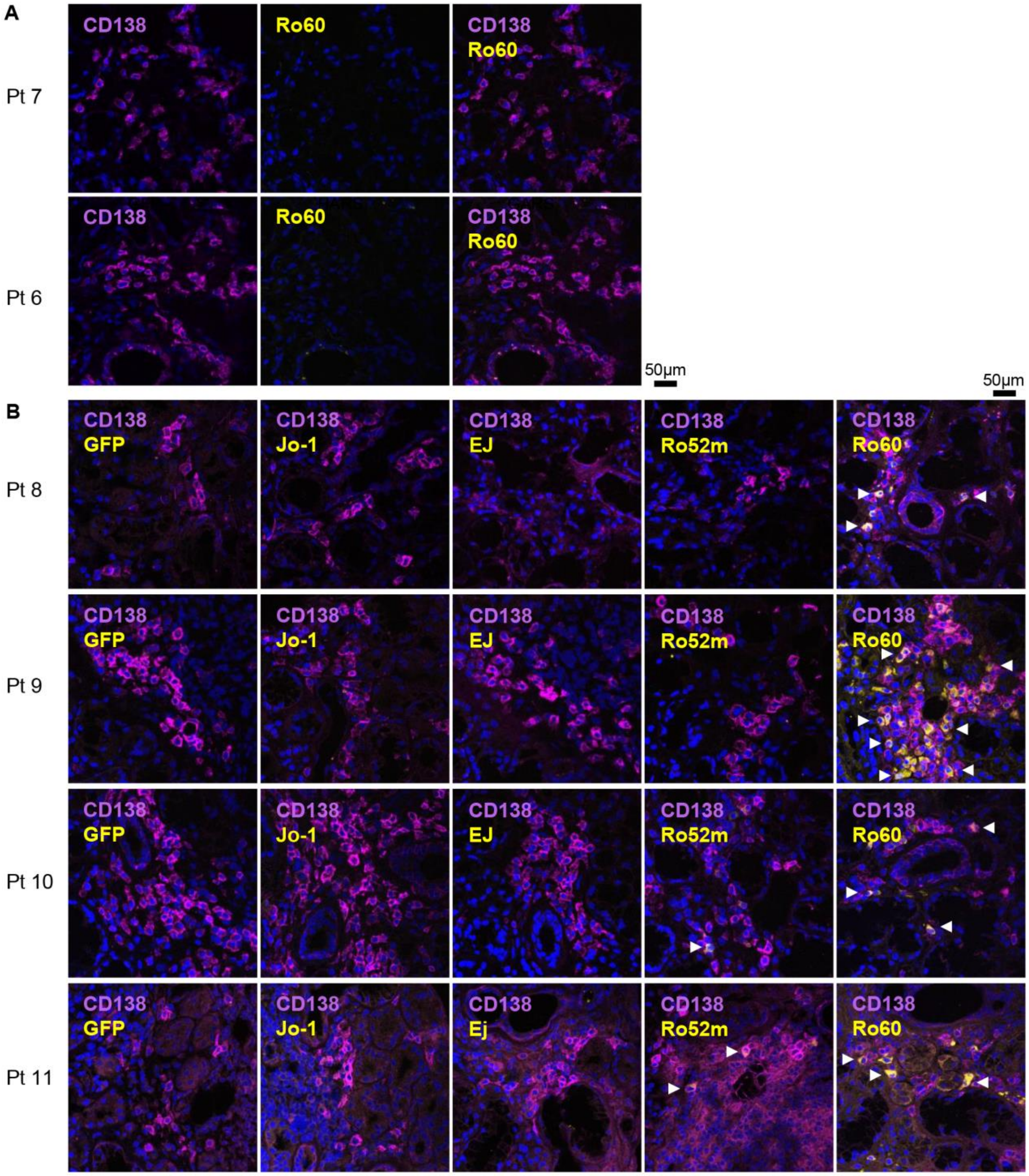

Supplementary Figure 4. Detection of autoantibody-producing cells by immunofluorescence using autoantigens

Fresh-frozen sections of salivary glands were stained with purified GFP or GFP-autoantigen fusion proteins, anti-CD138 antibody (a marker of the antibody-producing cells), and DAPI. (A) Representative single marker and overlay images of the anti-Ro60 antibody–producing cells in salivary glands from patient 6 and patient 7 are shown. (B) Representative overlay images of the anti-Jo-1, anti-EJ, anti-Ro52m, and anti-Ro60 antibody–producing cells in salivary glands from serum anti-Ro60 antibody–positive SjS patients are shown. White arrowheads indicate autoantibody-producing cells. Scale bar indicates 50 μm.

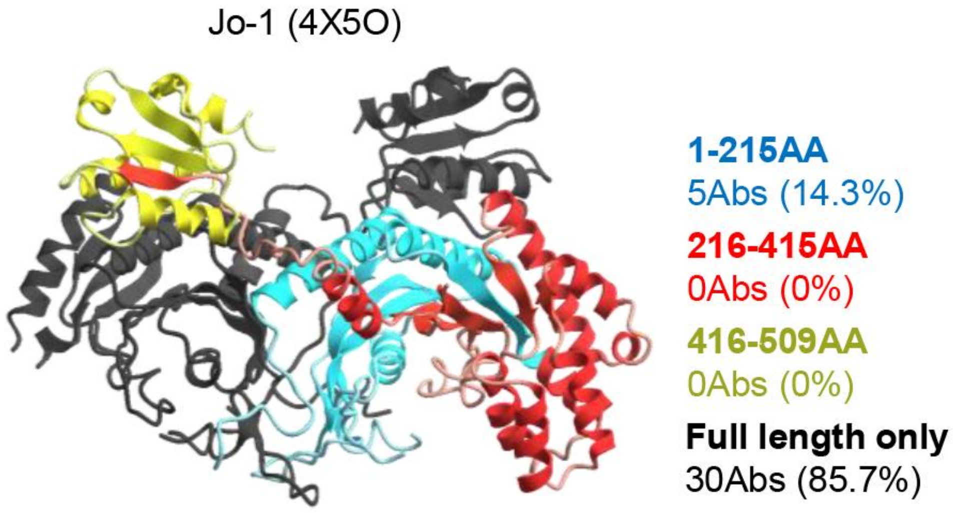

Supplementary Figure 5. Binding mode of anti-Jo-1 antibody

The reactivities of autoantibodies to full-length or fragmented Jo-1-binding beads were examined. The proportions of antibodies that can recognize each fragment (red, blue, and yellow) or only the full-length Jo-1 (black) are shown. The figure shows the three-dimensional structures of Jo-1 (4X5O), colored by the fragments. Note that the reported structure does not include the positions of some amino acids.

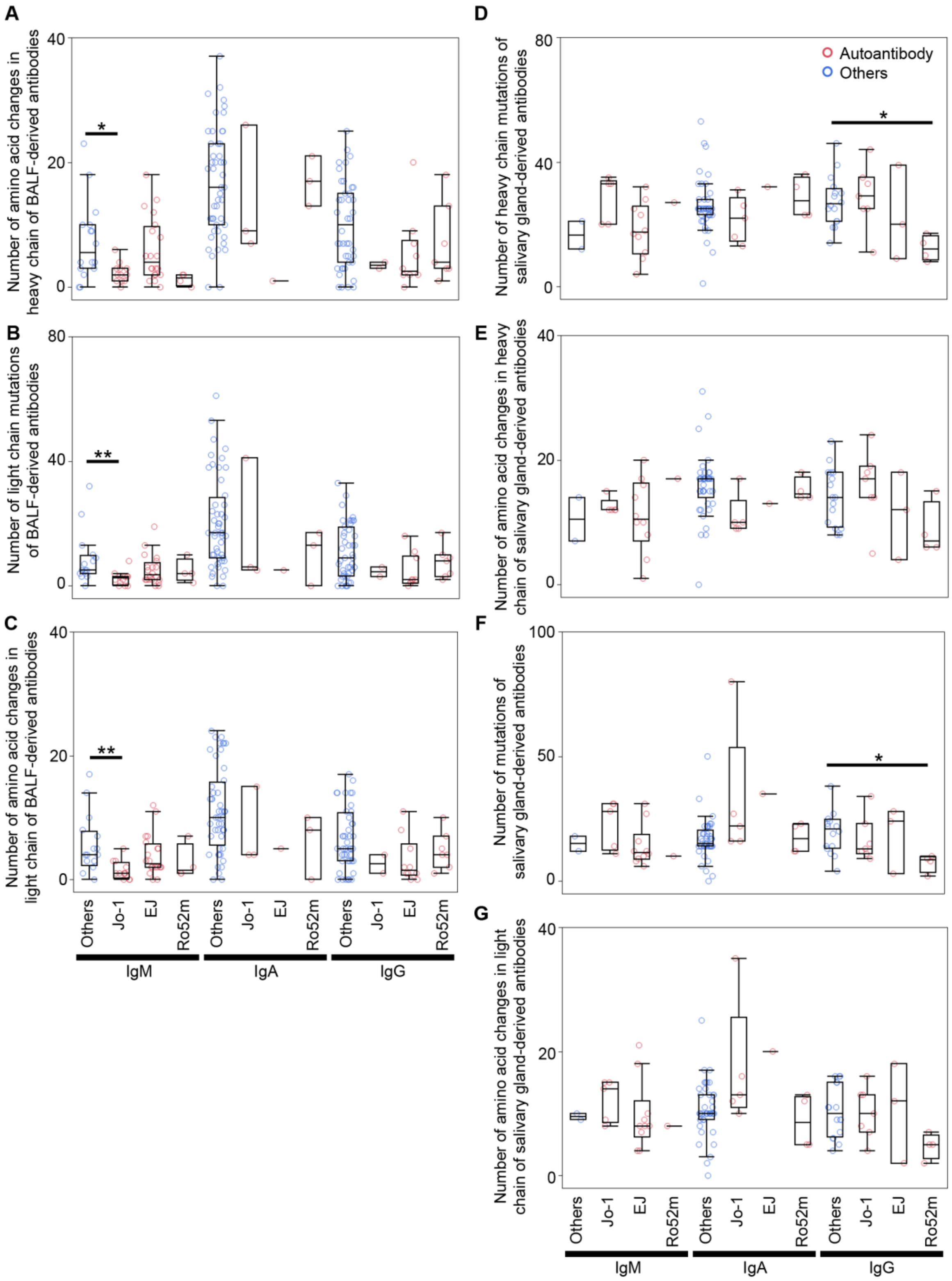

Supplementary Figure 6. Characteristics of autoantibodies in ASS patients

The number of the somatic hypermutations and amino acid changes of heavy and light chain of the antibodies from BALF samples were compared between autoantibodies and other antibodies by Steel test. *p < 0.05, **p < 0.01.

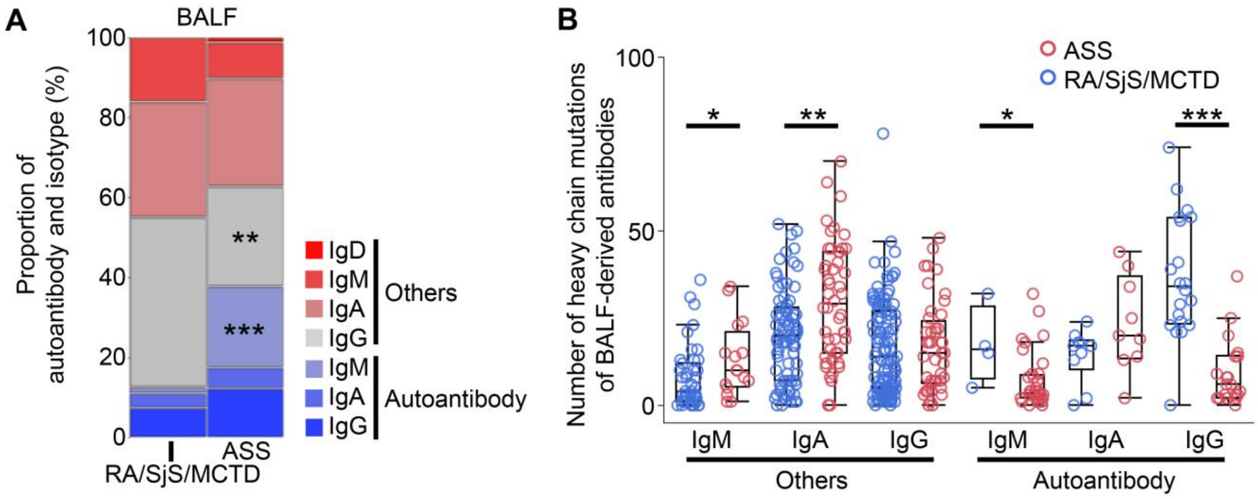

Supplementary Figure 7. Comparison of isotype and somatic hypermutations of antibodies produced in this study and previous study.

(A) The autoreactivity and isotypes of the antibodies identified in this study were compared with previous studies. **$p < 0.01$, ***$p < 0.001$ by Fisher's exact test with Bonferroni correction. (B) The somatic hypermutations of heavy chain of BALF-derived antibodies in this study were compared with previous study (22). Wilcoxon test. *$p < 0.05$, **$p < 0.01$, ***$p < 0.001$.

1.2 Supplementary Table

Supplementary Table 1. Clinical characteristics of SjS patients for salivary gland analysis

	Pt8	Pt9	Pt10	Pt11
Age	44	51	44	36
Sex	F	F	F	F
2016 ACR/EULAR criteria for SjS	Fulfilled	Fulfilled	Fulfilled	Fulfilled
Other autoimmune diseases	-	-	-	-
Disease duration (months)	41	120	60	1
ANA titer	640	160	160	640
ANA type	s	h, s	s	s
Serum anti-Ro60 antibody titer[a]	574	>1200	>1200	>1200
Serum anti-SSB antibody titer[a]	>1200	neg	neg	>1200
Medication	no	no	no	no
Gum test (ml/10 min)	6.0	10.0	5.3	0.6
Schirmer test, right, left (mm/5 min)	3, 3	NA	0, 0	13, 9
SPK	negative	negative	positive	positive
Focus score	<1	1	>1	>1

[a]From medical records, measured by chemiluminescence enzyme immunoassay.

SjS: Sjögren's syndrome, ANA: anti-nuclear antibody test (s: speckled, h: homogenous), NA: not assessed, SPK: superficial punctate keratopathy.
